# Balancing Mass Transfer and Active Sites to Improve Electrocatalytic Oxygen Reduction by B,N Codoped C Nanoreactors

**DOI:** 10.1021/acs.nanolett.3c00202

**Published:** 2023-03-23

**Authors:** Xuefei Wang, Tianyi Liu, Haitao Li, Chao Han, Panpan Su, Na Ta, San Ping Jiang, Biao Kong, Jian Liu, Zhenguo Huang

**Affiliations:** † State Key Laboratory of Catalysis, Dalian Institute of Chemical Physics, Chinese Academy of Sciences, 457 Zhongshan Road, Dalian 116023, China; § School of Civil & Environmental Engineering, University of Technology Sydney, Sydney, New South Wales 2007, Australia; ζ Department of Chemistry, Shanghai Key Lab of Molecular Catalysis and Innovative Materials, Collaborative Innovation Center of Chemistry for Energy Materials, Fudan University, Shanghai 200438, China; ‡ DICP-Surrey Joint Centre for Future Materials, Department of Chemical and Process Engineering, University of Surrey, Guildford, Surrey GU2 7XH, United Kingdom; ⊥ School of Materials Science and Engineering, Central South University, Changsha 410083, China; # Department of Minerals, Energy and Chemical Engineering, Fuels and Energy Technology Institute & WA School of Mines, Curtin University, Perth, Western Australia 6102, Australia; △ Yiwu Research Institute of Fudan University, Yiwu, Zhejiang 322000, China

**Keywords:** oxygen reduction, nanoreactor, mass transfer, electrocatalysis

## Abstract

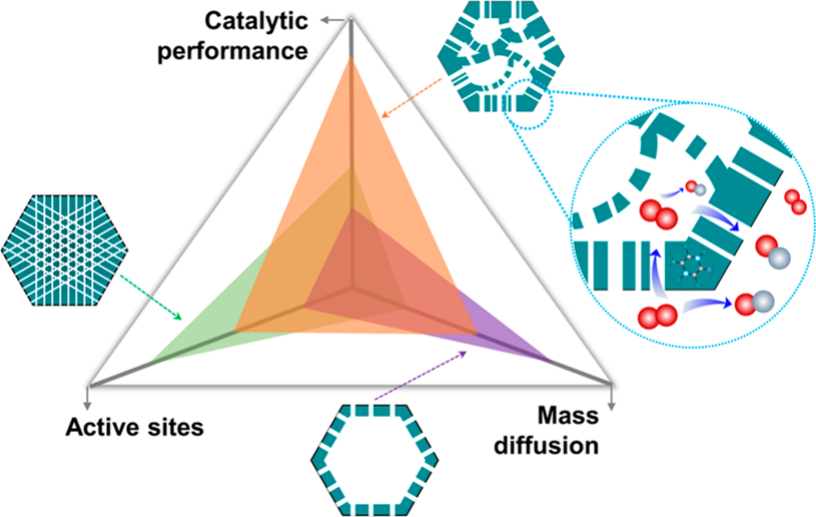

Mass transfer is critical in catalytic processes, especially when the reactions are facilitated by nanostructured catalysts. Strong efforts have been devoted to improving the efficacy and quantity of active sites, but often, mass transfer has not been well studied. Herein, we demonstrate the importance of mass transfer in the electrocatalytic oxygen reduction reaction (ORR) by tailoring the pore sizes. Using a confined-etching strategy, we fabricate boron- and nitrogen-doped carbon (B,N@C) electrocatalysts featuring abundant active sites but different porous structures. The ORR performance of these catalysts is found to correlate with diffusion of the reactant. The optimized B,N@C with trimodal-porous structures feature enhanced O_2_ diffusion and better activity per heteroatomic site toward the ORR process. This work demonstrates the significance of the nanoarchitecture engineering of catalysts and sheds light on how to optimize structures featuring abundant active sites and enhanced mass transfer.

Carbon-based metal-free catalysts with stable structures, rich defects, and tunable electronic structures have attracted much attention for various chemical reactions, including thermocatalytic, electrocatalytic, and photocatalytic reactions.^1,2^ In the past few decades, numerous efforts have been devoted to optimizing the electronic structure of active centers at the nanoscale by heteroatom doping, especially with multiple heteroatoms.^3^ Among these, boron and nitrogen codoped carbon (B,N@C) has displayed an interesting catalytic performance.^4−6^ Most of the studies reported to date have focused on tuning chemical compositions and nanostructures to improve the efficacy and quantity of active sites.

An often less studied but significant factor affecting the catalytic process is mass transfer, which determines the efficiency of the supply of reactants and removal of products to and from the active sites and consequently the catalytic performance. For various gas-involving electrocatalysis, including hydrogen evolution reaction (HER), oxygen evolution reaction (OER), oxygen reduction reaction (ORR), electrochemical reduction of carbon dioxide (CO_2_RR), and electrochemical reduction of nitrogen (N_2_RR), the diffusion behavior of reactants and products plays a practical role in these heterogeneous catalysis process. So far, electrode fabrication and device configuration design have been the common approach to enhance mass transfer,^7−10^ rather than tailoring the pore sizes and volumes of the nanocatalyst itself.^11,12^

Nanoreactors are effective platform materials with a wide range of structures where confined environments can modulate chemical reactions.^13,14^ Trimodal porous nanoreactors with micropores (<2 nm), mesopores (2–50 nm), and macropores (>50 nm) are desirable to simultaneously achieve large numbers of active sites and improve mass diffusion.^15−18^ Metal–organic frameworks (MOFs) are ideal precursors to obtain nanoreactors due to their customizable modular assembly and controllable morphologic and structural evolution.^1,19−22^ Key pore parameters including pore shapes, sizes, and volumes can be regulated by controlling etching conditions. By varying the etching time, etching temperature, and etchant concentration, mesopore sizes were confined within a range of 2–38 nm.^23−25^ However, the range of the pore sizes is not wide enough. Furthermore, pyrolyzing zinc-based MOFs (Zn-MOFs) has been used to obtain porous metal-free carbon materials with heteroatomic active sites.^26^ However, most of these active sites are deeply hidden in the micropores of MOFs-derived nanocarbons and are unreachable for the catalytic reaction.^27^ Selective etching of MOFs followed by pyrolysis proves to be effective in obtaining tunable nanostructures where active sites are exposed to reactants.^28,29^ However, synthesizing a series of platform materials with different porous structures but similar active sites in efficacy and quantity has been far less developed. Therefore, the relationship between mass transfer and catalytic activity is not well understood. B,N@C nanostructures have been derived by directly pyrolyzing B,N-containing MOF (e.g., boron imidazolate framework (BIF)-82^30^ and BIF-1S^31^) or MOFs mixed with B-containing substances.^6,32−40^ Most of these MOF-derived B,N@C nanomaterials exhibit collapsed structures and narrow pore size distributions.^34−40^ To obtain a great variety of pores with good controllability, this area needs to be studied deeply, including selecting the right precursors and the pyrolysis conditions.

Herein, we developed an effective method to fabricate porous three-dimensional (3D) B,N@C catalysts by sequentially etching and pyrolyzing a series of ZIF-8 precursors. It is known that most of the reported etching processes are too fast to precisely regulate the porosity of the MOF precursors.^28,41^ For example, tannic acid can transfer solid ZIF-8 and NH_2_-MIL-125(Ti) into hollow MOFs in a very short period (<30 min).^41^ To address this issue, ammonia borane (AB), which features mild hydrolysis, was used to make the etching process controllable. Consequently, the structure of the B,N@C nanocages was effectively tailored. These 3D B,N@Cs feature similar catalytic active sites for ORR, in terms of chemical composition and quantity. But their performance correlates with the porous structure, especially at the meso- and macroscale, which affects the mass transfer during the heterogeneous process. The performance of the optimized B,N@C-24 catalyst is on par with commercial Pt/C, and the excellent catalysis is associated with the trimodal-porosity, enabling the best combination of active site exposure and mass transfer.

The synthesis of the B,N@C nanoreactors is illustrated in Figure 1. A double-solvent method was first used to immobilize ammonia borane (AB) as guest molecules in the ZIF-8 hosts on account of the immiscibility between water and cyclohexane. AB provides B and N as dopants and acts as a mild etching agent by releasing protons during its slow hydrolysis, i.e., NH_3_BH_3_ + 4H_2_O → NH_4_^+^ + B(OH)_4_^–^ + 3H_2_.^29,42^ Therefore, compositional and structural modifications of ZIF-8 hosts were achieved at the same time, producing AB@ZIF-*x*h particles, where *x* represents the etching time. In the subsequent pyrolysis step, AB@ZIF-*x*h was transformed to corresponding B,N@C-*x*h nanocages with hierarchical porosities.^43^

**Figure 1:**
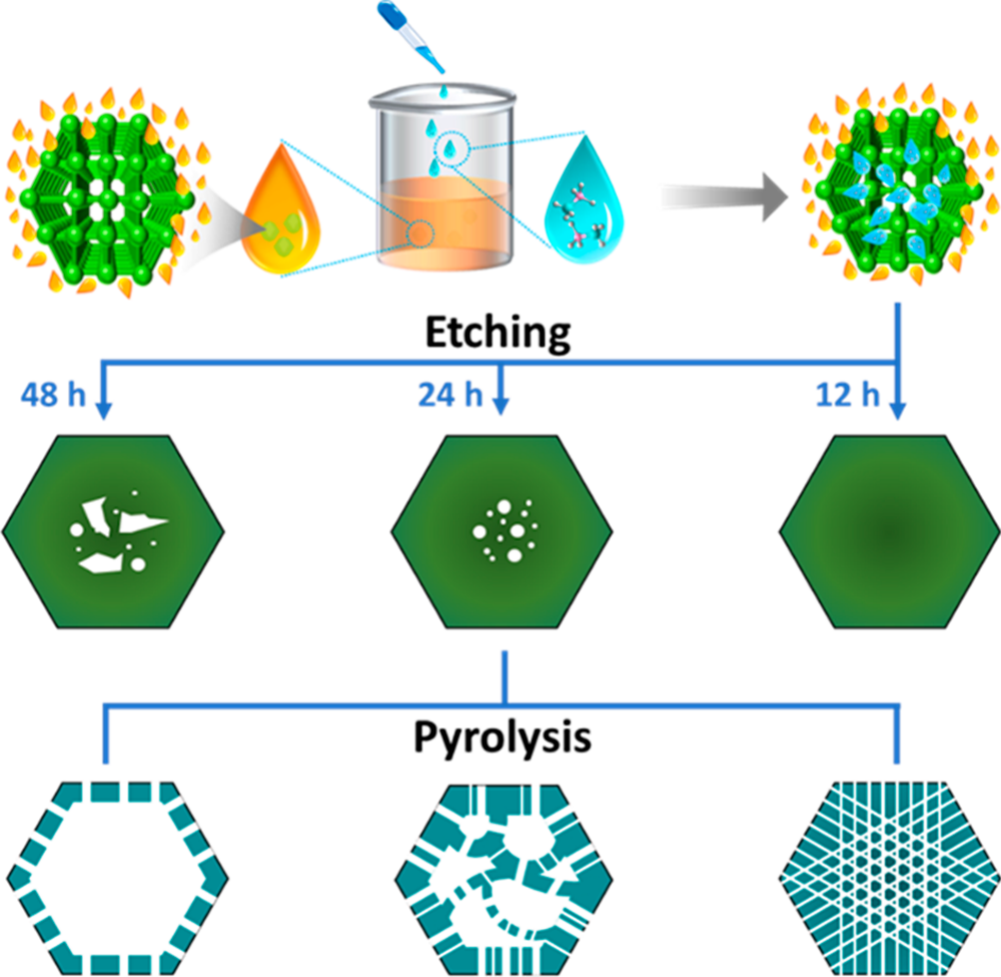
Schematics of the preparation of the B,N@C nanoreactors.

The X-ray diffraction (XRD) patterns of the resulting AB@ZIF-8 match the simulated powder XRD patterns based on the ZIF-8 single crystal (Figure S1). The scanning electron microscopy (SEM) images show negligible changes in morphologies and sizes after etching (Figure S2). Transmission electron microscopy (TEM) images reveal the structural evolution of the AB@ZIF-8 nanoparticles through etching (Figure 2a–c and Figure S3). AB@ZIF-2/6/12h nanoparticles retain the solid rhombic dodecahedron structure without obvious pores as the parent ZIF-8 (Figure 2a and Figure S3). Extended etching gradually led to the formation of larger pores inside the host (Figure 2b, c). EDS mapping of AB@ZIF-24h in Figure 2d revealed that N and C were uniformly dispersed in the whole nanoparticle, while B and O were concentrated to the center of the particles, indicating that AB molecules were diffused inward through capillary action and hydrolyzed inside the host. These results prove that confined etching is effective in structurally modifying the MOF hosts.

**Figure 2:**
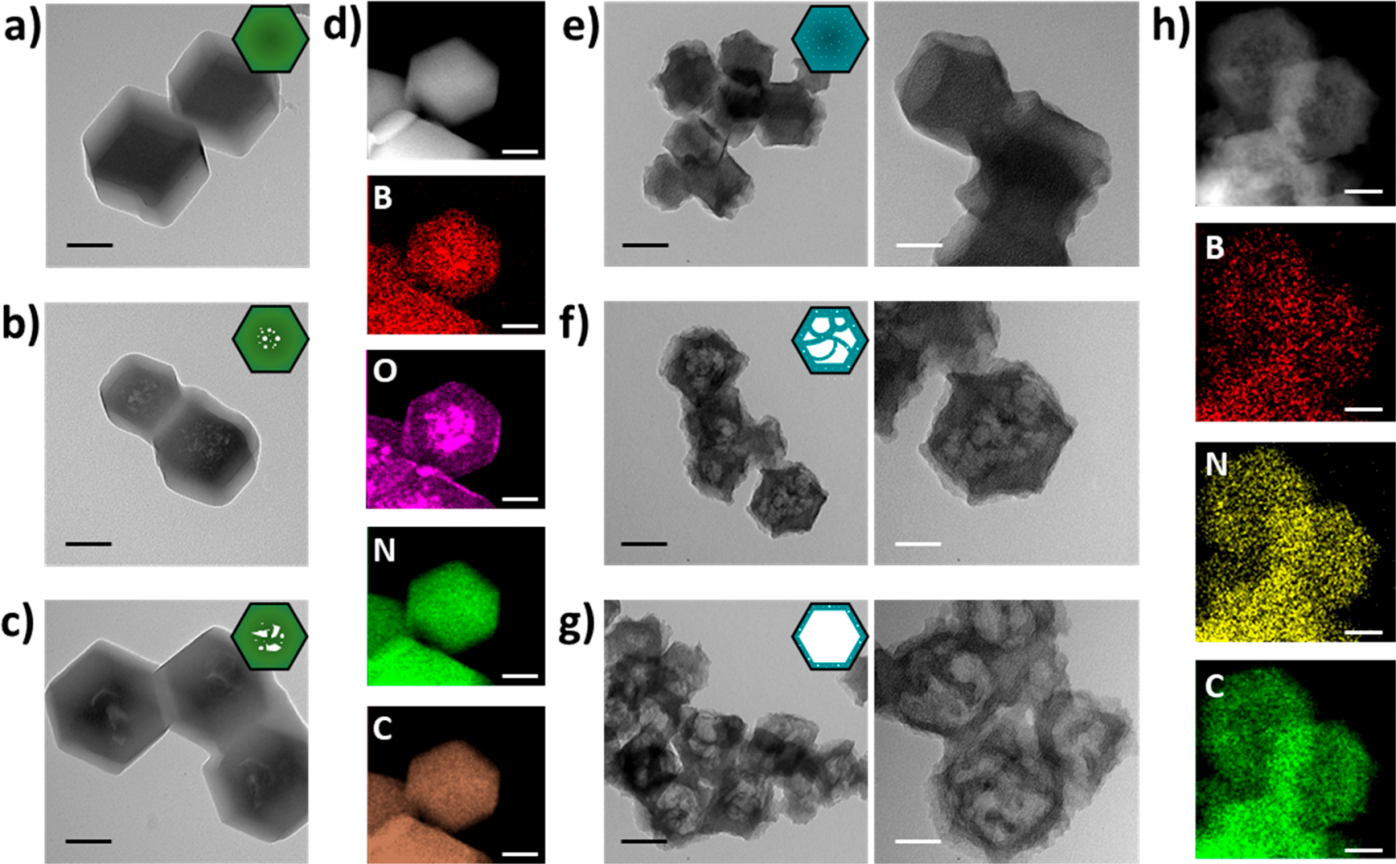
Controllable etching of ZIF-8 precursors and derived hierarchical porous B,N@Cs. TEM images of (a) AB@ZIF-12h, (b) AB@ZIF-24h, (c) AB@ZIF-48h, (e) B,N@C-12h, (f) B,N@C-24h, and (g) B,N@C-48h. EDS mapping of (d) AB@ZIF-24h and (h) B,N@C-24h. Black and white scale bars are 100 and 50 nm, respectively.

Subsequently, B,N@C nanoreactors with different architectures were prepared by pyrolyzing the AB@ZIF-8 precursors at 1000 °C under N_2_. As illustrated by SEM images in Figure S4a, B,N@C-12h retains a polyhedral morphology similar to that of the AB@ZIF-12h precursor after pyrolysis. With the extension in etching, B,N@C became hollow and porous with rough surfaces collapsed inward (Figure S4b, c). TEM images further verify the correlation between the nanoarchitecture of B,N@C (Figure 2e–g), and AB@ZIF-8 precursors (Figure 2a–c). Specifically, the greater degree to which the precursor was etched, the larger the cavities of the derived materials. As a result, B,N@C-12h possesses small voids inside; B,N@C-24h transforms into nanocages with multiple compartments; and B,N@C-48h becomes hollow cages. Elemental mapping images reveal that B and N heteroatoms are homogeneously distributed together with C in all these structures (Figure 2h), indicating the formation of uniform codoping of C by B and N.

To further explore the structural dependency of B,N@C on AB@ZIF-8, their structures were evaluated using N_2_ adsorption–desorption analysis (Figure 3a, b). The sorption isotherms of the AB@ZIF-8 precursors (Figure 3a) display representative type I isotherms, indicating the dominance of micropores in these materials. The specific surface area decreased with longer etching, meaning that etching transformed some micropores into mesopores or macropores.^44^ The pore size distribution profiles calculated based on nonlocal density functional theory (NLDFT) in Figure S5 confirmed the decrease in micropore (<2 nm) proportions and the increase in mesopore (2–50 nm) and macropore (>50 nm) proportions during etching. N_2_ adsorption–desorption isotherms were also collected to characterize the porosities of B,N@C nanocages (Figure 3b). Different from AB@ZIF-8, B,N@C exhibited a combination of type I and IV isotherms with clear hysteresis loops, indicating the existence of micro-, meso-, and macropores.^18,45^ There are several possible mechanisms for the formation of porous B, N@C nanocages, which include the generation of gases,^46^ the evaporation of Zn atoms,^47^ and the generation of graphitic structure.^28^ All of the samples have undergone the same pyrolytic conditions, so the differences in porous structures of B,N@C nanocages are mainly derived from the difference among the AB@ZIF-8 precursors.

**Figure 3:**
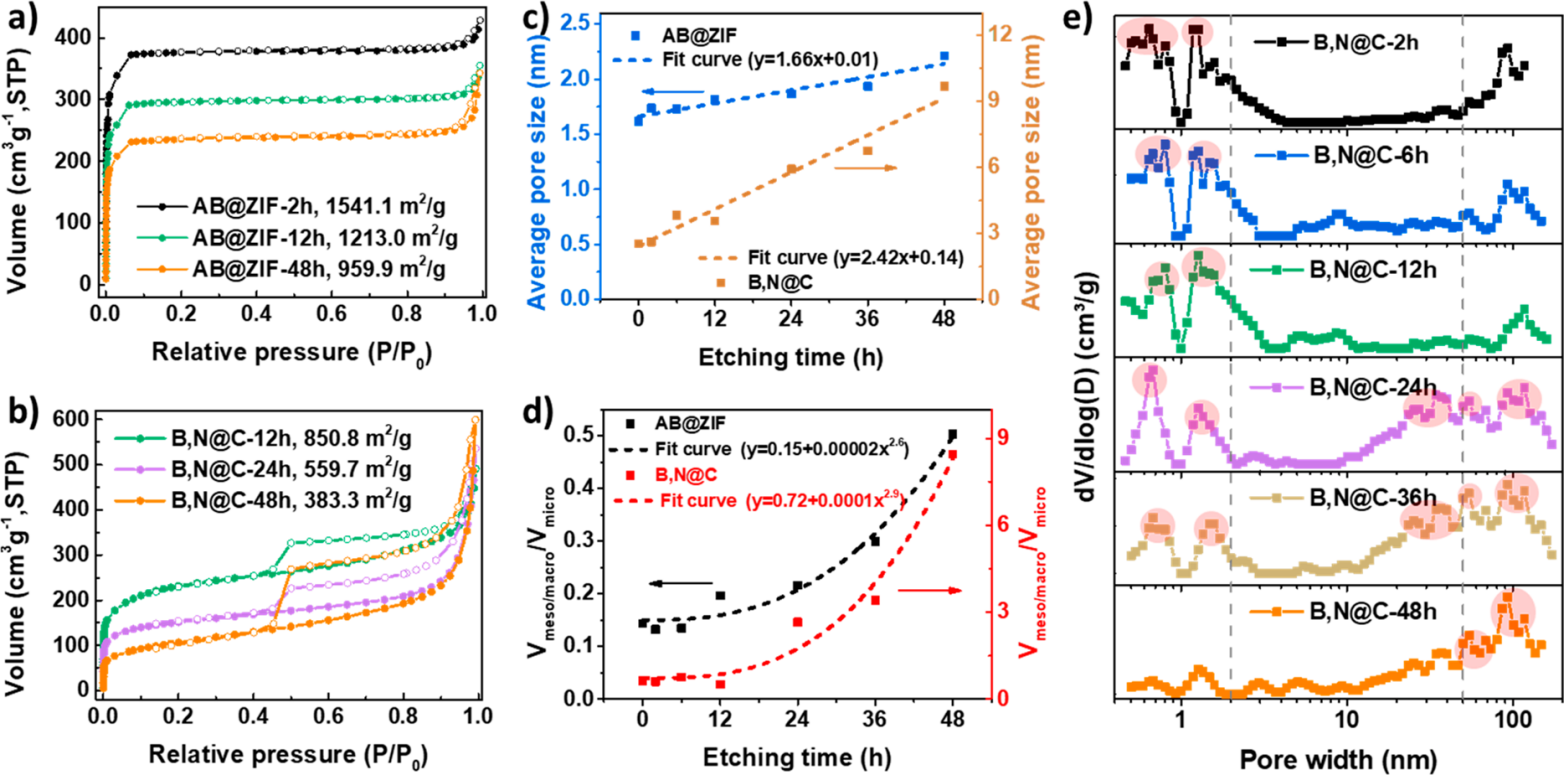
Structural characterization of AB@ZIF-8 nanoparticles and B,N@C nanocages. N_2_ adsorption–desorption isotherms of (a) AB@ZIF-8 and (b) B,N@C, (c) average pore size, and (d) pore volume ratios of meso/macro to micropore in AB@ZIF-8 and B,N@C, and (e) pore size distributions of B,N@C.

To study the correlation of the porosity between AB@ZIF-8 nanoparticles and B,N@C nanocages, the relationship between their pore sizes and etching time was studied based on their N_2_ adsorption–desorption curves (Figure 3a, b and Figure S6). Figure 3c presents plots of average pore size versus etching time for AB@ZIF-8 and B,N@C, and the relationship between these two is based upon curve fitting. For both AB@ZIF-8 and B,N@C, there is a linear correlation between the average pore size and etching time. The ratios of meso/macroporous to microporous volumes of AB@ZIF-8 precursors with etching time were also analyzed (Figure 3d). The difference in the first 6 h is minimal, while the ratio increases faster after 12 h, approximating to a power function relationship. This agrees well with the TEM images and confirms the essential role of AB in the host–guest chemistry-assisted etching to structurally modify the host, so that B,N@C with tunable structures can be obtained. The ratio of meso/macroporous to microporous volumes of B,N@C nanocages shows a similar to that of AB@ZIF-8 and can be fitted to the power function with a similar power. These results bring out the feature of MOF-derived carbon, i.e., its architectures depending on the MOF precursors. However, most of the reported etching processes are too fast to precisely regulate the porosity of the parent MOFs. For example, tannic acid changed solid ZIF-8 and NH_2_-MIL-125(Ti) into hollow MOFs in a very short time (<30 min).^28,41^ In contrast, AB features mild hydrolysis, which makes the etching process moderate and controllable. Consequently, the structure of the B,N@C nanocages can be effectively tailored.

The NLDFT pore size distribution analysis (Figure 3e) shows that B,N@C-2/6/12h samples mainly possess micropores with sizes around 0.7 and 1.3 nm. B,N@C-24/36h samples present mesopores at about 25.3 and 37.1 nm, macropores at 93.1 and 117.2 nm, together with micropores at 0.7 and 1.3 nm, confirming them to be trimodal-porous. B,N@C-24h has a more balanced micro-, meso-, and macropores than that of B,N@C-36h. Macropores with the size of 54.4, 93.1, and 117.2 nm dominated in B,N@C-48h. These results indicate that B,N@C-12/24/48h possess different nanoarchitectures, rendering them ideal material platforms to test the impact of porosity on mass transfer and consequent ORR performance.

B,N@C-12/24/48h was further investigated using XRD, Raman spectroscopy, and X-ray photoelectron spectroscopy (XPS) techniques. XRD patterns in Figure 4a show broad diffraction peaks at 25°, which could be indexed as the (002) crystal plane of amorphous carbon.^48^ Raman spectra (Figure 4b) can be deconvoluted into four types of carbon configurations at 1348 (D_1_), 1119 (D_2_), 1471 (D_3_), and 1577 (G) cm^–1^. D_1_, D_2_, and D_3_ bands correspond to the disordered carbon structure, amorphous carbon, and carbon atoms outside of a perfectly planar graphene network, respectively, while the G band is attributed to the ordered graphite carbon.^49,50^ The *I*_D1_/*I*_G_ values for all of the B,N@C nanoreactors are similar, within the range of 1.44 to 1.54, indicating their similar degree of graphitization and defects. Note that the D_2_ and D_3_ bands contribute less to the ORR performance.^49^ Chemical compositions and chemical states were analyzed with XPS. Survey scans show that all of these catalysts contain B, C, N, and O (Figure S7 and Table S1). High-resolution XPS spectra of B 1s can be deconvolved into three peaks, which are assigned to B–C (190.6 eV), B–N (191.9 eV), and B–O (192.5 eV) (Figure 4c and Table S2). It is notable that, by prolonging the etching time, the proportion of B–O increased significantly from 0% in B,N@C-12h to 23% in B,N@C-48h, indicating the gradual accumulation of B–O during etching. This result matches well with our proposed etching mechanism in which the mild hydrolysis of AB gradually releases protons, which then attack 2-MIM ligands in ZIF-8. For ORR, B–O bonds are reported to have a rather limited contribution to the performance,^51^ where B–C and B–N benefit the ORR.^52,53^ The N 1s XPS spectra are well fitted by five peaks corresponding to N–B (397.9 eV), pyridinic N6 (398.5 eV), pyrrolic N5 (399.5 eV), graphitic N (400.8 eV), and N–O (402.4 eV). Pyridinic N6, pyrrolic N5 and graphitic N are also known to contribute to ORR.^54,55^ The ratios of B (in B–N) to N species (in N–B) are similar in these catalysts (2.4 for B,N@C-12h, 2.5 for B,N@C-24h, and 2.5 for B,N@C-48h), but the total amounts of B and N in B,N@C-24h are slightly lower compared with the other two samples (Table S2). As shown in Figure 4d and Table S2, all these catalysts have similar chemical features of N and all these known active sites involving B and N are similar in ratio. Therefore, these three catalysts with similar heteroatomic B_2.5_–N_1_ motifs can serve as an ideal material platform to investigate the structure-performance relationship of nanoreactors toward catalytic reactions.

**Figure 4:**
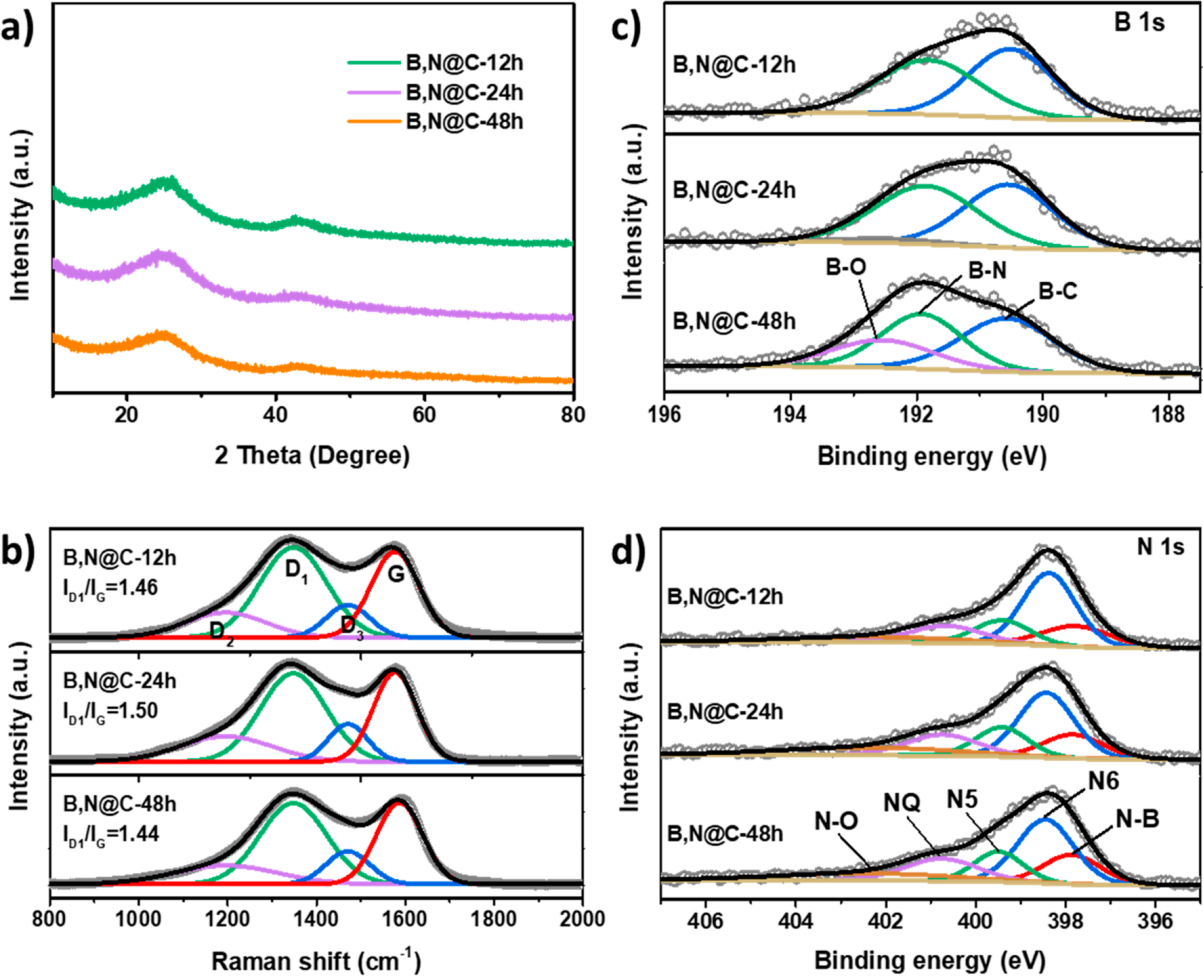
Characterization of the B,N@C nanoreactors. (a) PXRD patterns, (b) Raman spectra, (c) XPS spectra of B 1s, and (d) XPS spectra of N 1s.

Electrochemical ORR was used as a model reaction to test the effect of porous structural engineering on the catalytic performance and to understand the importance of mass transfer during the reaction. The performance was assessed by using a rotation ring disk electrode (RRDE) in a typical three-electrode system. The LSV disk curves collected at 1600 rpm in an O_2_-saturated 0.01 M (pH 12) and 0.1 M (pH 13) KOH solution are shown in Figure 5a and Figure 5b, respectively. Generally, dilute electrolytes exhibit low viscosity, therefore, having negligible effects on mass diffusion. As a result, the active site determines the electrocatalytic performance. As can be seen from Figure 5a, the difference in diffusion-limited current density (DLCD) among the three samples is negligible in the 0.01 M KOH solution. As expected, B,N@C-12h with the largest surface area shows the most positive half-wave potential (*E*_1/2_) of 0.557 V vs RHE (Figure S8), while B,N@C-48h with the lowest surface area shows the most negative *E*_1/2_ of 0.524 V vs RHE. These results confirm that the electrocatalytic performance is determined by the exposure of active sites in dilute electrolytes, which is typically related to surface areas. In contrast, the ORR performance of the three samples in 0.1 M KOH varied greatly (Figure 5b and c). It is known that when the electrolyte concentration increases, the viscosity of the electrolyte increases correspondingly^56^ and the effect of mass transfer on catalytic performance becomes more significant. Therefore, the catalytic activity was determined by both the exposure of the active sites and the mass transfer efficiency. As shown in Figure 5c, B,N@C-24h with the most balanced trimodal-porous structure among the three catalysts (Figure 5d), achieved the largest diffusion-limited current density (DLCD) of −5.9 mA cm^–2^ at 0.2 V vs RHE. In addition, B,N@C-24h catalyst featured the most positive onset potential (*E*_on_) of 0.979 V and *E*_1/2_ of 0.861 V vs RHE, as summarized in Figure 5c. The better ORR performance of B,N@C-24h than B,N@C-12h in 0.1 M KOH is mainly due to the enhancement of mass transfer in meso/macropores compared with micropores, demonstrating that mass transfer is vital for ORR in concentrated electrolyte solutions. Meanwhile, B,N@C-48h shows the low activity among these three catalysts, which indicates that mass transfer is not the only important factor in determining performance.

**Figure 5:**
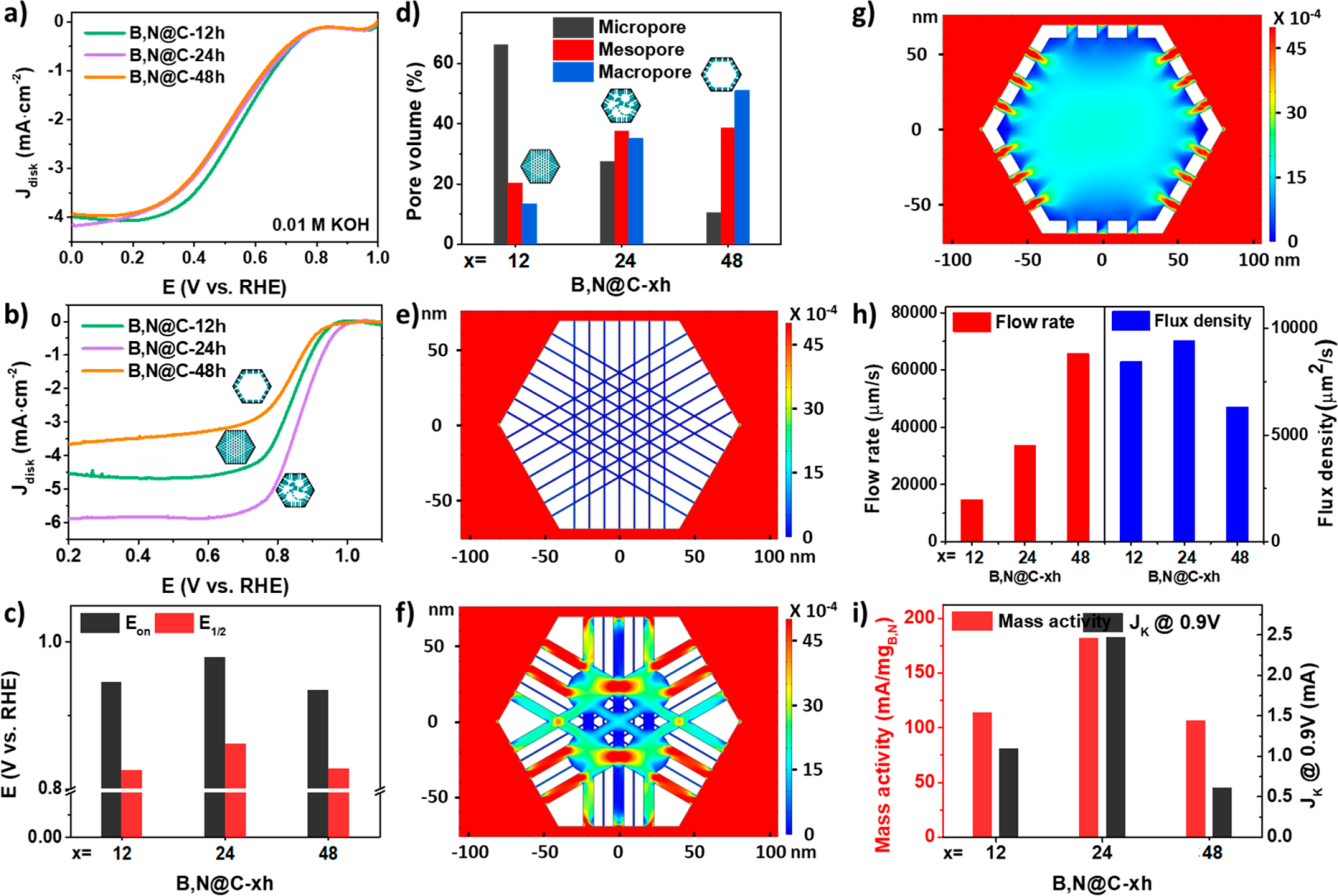
Electrocatalytic ORR performance of the B,N@C nanoreactors. (a) LSV curves in 0.01 M KOH; (b) LSV curves and (c) corresponding onset and half-wave potentials in 0.1 M KOH; (d) relative pore portions. Velocity fields of nanoarchitecture models: (e) B,N@C-12h, (f) B,N@C-24h, and (g) B,N@C-48h; (h) the simulated flow rate and flux density for B,N@C-12/24/48h, and (i) mass-specific activities and kinetic current densities.

Finite element analysis (FEA) simulations were carried out to further understand the influence of mass transfer on the electrocatalytic performance. According to the structural characterizations (Figure 2 and 5d), the main difference between the three samples is the pore sizes and volumes in the nanoreactor. Models were constructed to reflect the cavity size and porous channels. As shown in Figure 5e–g, the models exhibit micropore-dominated, balanced trimodal-porous, and macropore-dominated structures, corresponding to the experimentally synthesized B,N@C-12h, B,N@C-24h, and B,N@C-48h, respectively. The different colors in Figure 5e–g are associated with the velocity of simulated flow fields. The flow velocity, which is significantly influenced by the geometric architectures of the materials, is an important index to evaluate mass diffusion. As expected, the flow rate in the macropore-dominated hollow cage is overall larger than that in the micropore-dominated and balanced trimodal-porous structures (Figure 5h), with only a few positions having rates similar to those of the trimodal-porous structures (Figure 5f, g). The electrochemical ORR process is mainly determined by the accessibility of active sites, which is affected not only by the mass transfer efficiency but also by the exposure of active sites. Therefore, the flux passing the entire internal surface was introduced (denoted as flux density hereafter), which takes into account both the active site exposure affected by the specific surface areas and the mass transfer determined by the porous structure. The flux density helps to investigate the process intensification by the porous structural engineering of nanoreactors. The micro/meso/macropore-balanced model has a clear advantage over the other two models (Figure 5h). The flux density decreases significantly in the macropore-dominated hollow cage, because of the limited surface area. Meanwhile, the flux density of the micropore-dominated sample is also smaller than that of the trimodal-porous structure because of the limited mass transfer. On all of these counts, a trimodal-porous architecture is considered to be the desired nanoreactor to boost the process intensification.

As discussed above, B,N@C-24h with trimodal-porous structure delivered the largest *E*_on_, *E*_1/2_, and DLCD among these three catalysts, and its performance is comparable to that of commercial Pt/C (Figure S9), which shows an *E*_on_ of 1.01 V, an *E*_1/2_ of 0.867 V, and a DLCD of −5.6 mA cm^–2^ at 0.2 mV vs RHE. Besides, the performance of B,N@C-24 nanoreactors is on par or even better than the reported metal-free carbon-based catalysts (Table S3). In contrast, micropore-dominated B,N@C-12h demonstrated lower *E*_on_, *E*_1/2_, and DLCD than B,N@C-24h, although it has the highest heteroatom content (Table S1). This suggests that certain active sites in the micropores have no contact with the reactants, so the ORR rate is limited.^57^ In addition, B,N@C-48h with a larger portion of macropores also shows decreased ORR activities compared with those of B,N@C-24h (Figure 5b, c), which is mainly due to reduced surface active sites. To further test the effect of porous structural engineering on process intensification toward the ORR, heteroatomic mass activity and kinetic current density (*J*_K_) were calculated. B,N@C-24h with trimodal-porous distributions delivers the highest mass-specific activity and *J*_k_ among the three catalysts (Figure 5i). In addition, B,N@C-24h possesses the lowest Tafel slope (Figure S10), further manifesting that the trimodal-porous nanoreactor exhibits the fastest kinetics toward the ORR. All these results prove that a trimodal-porous nanoreactor is desired for improving both mass transfer and exposure of active sites. Based on RRDE measurement (Figure S11a and Figure 5b), the average electron transfer number (*n*) of B,N@C-24h was calculated to be about 3.83 (Figure S11b), indicating an efficient 4e^–^ pathway toward ORR, which is desirable for zinc-air batteries. Similarly, electron transfer numbers were found for B,N@C-12h and B,N@C-48h.

We designed a series of hierarchical porous carbon nanoreactors doped with B and N via efficient confined-etching and pyrolysis of ZIFs. The B,N@C nanocages have similar catalytic active sites in terms of intrinsic activity and quantity but have different pores in terms of size and volume. When tested for the ORR, the B,N@C nanoreactor with abundant micro-, meso-, and macropores shows the highest catalytic activity. Experimental results and FEA calculations confirm that such a trimodal-porous architecture enhances process intensification toward ORR because of enhanced mass transfer and effective active site exposure, in comparison with microporous and macroporous architectures. This work demonstrates the importance of mass transfer during heterogeneous catalysis, which should be considered when designing novel catalysts. It also proves the efficacy of the host–guest chemistry-assisted etching strategy, especially with mild etchants, for the synthesis of heteroatom-doped carbon nanoreactor systems.

## Supporting Information Available

Material, experimental and simulation details, additional characterization, and electrochemical performances (PDF)
