# Supplementary material for: Balancing Mass Transfer and Active Sites to Improve Electrocatalytic Oxygen Reduction by B,N Codoped C Nanoreactors
Source: Nano Lett. 2023 Mar 23;23(11):4699–707. doi: 10.1021/acs.nanolett.3c00202 (PMC10273464; doi:10.1021/acs.nanolett.3c00202)
Supplement: Supplementary file 1 — nl3c00202_si_001.pdf [file nl3c00202_si_001.pdf]

Supporting Information for

Balancing mass transfer and active sites to improve electrocatalytic oxygen reduction by B,N co-doped C nanoreactors

Xuefei Wang,[†§] Tianyi Liu,[ζ‡] Haitao Li,[†] Chao Han,[⊥] Panpan Su,[†] Na Ta,[†] San Ping Jiang,[#] Biao Kong,[ζ] Jian Liu,[†,‡] and Zhenguo Huang[§*]*

[†] *State Key Laboratory of Catalysis, Dalian Institute of Chemical Physics, Chinese Academy of Sciences, 457 Zhongshan Road, Dalian 116023, China*

[§] *School of Civil & Environmental Engineering, University of Technology Sydney, Sydney, NSW, 2007, Australia*

[ζ] *Department of Chemistry, Shanghai Key Lab of Molecular Catalysis and Innovative Materials, Collaborative Innovation Center of Chemistry for Energy Materials, Fudan University, Shanghai 200438, China*

[‡] *DICP-Surrey Joint Centre for Future Materials, Department of Chemical and Process Engineering, University of Surrey, Guildford, Surrey GU2 7XH, UK*

[⊥] *School of Materials Science and Engineering, Central South University, Changsha 410083, China*

[#] *Department of Minerals, Energy and Chemical Engineering, Fuels and Energy Technology Institute & WA School of Mines, Curtin University, Perth 6102, Australia*

Yiwu Research Institute of Fudan University, Yiwu, Zhejiang 322000, China

Corresponding Author

Jian Liu, jianliu@dicp.ac.cn, jian.liu@surrey.ac.uk

Zhenguo Huang, zhenguo.huang@uts.edu.au

Supporting materials list

Figure S1. Powder X-ray diffraction (XRD) patterns of the as-synthesized AB@ZIF-8 precursors in comparison with simulated XRD patterns of ZIF-8 based upon single crystal XRD data.

Figure S2. SEM images of a) ZIF-8, b) AB@ZIF-12h, c) AB@ZIF-24h, and d) AB@ZIF-48h.

Figure S3. TEM images of a) ZIF-8, b) AB@ZIF-2h and c) AB@ZIF-6h.

Figure S4. SEM images of a) B,N@C-12h, b) B,N@C-24h, and c) B,N@C-48h.

Figure S5. Pore size distribution curves of AB@ZIF-8 precursors.

Figure S6. N_2 adsorption-desorption isotherms of a) AB@ZIF-8 and b) B,N@C.

Figure S7. XPS survey spectra of B,N@C.

Figure S8. Onset and half-wave potentials of the B,N@C nanoreactors in 0.01 M KOH solution.

Figure S9. LSV curves of commercial Pt/C in an O_2-saturated electrolyte (0.1 M KOH).

Figure S10. Tafel plots of the B,N@C nanoreactors in O_2-saturated electrolytes (0.1 M KOH).

Figure S11. a) The detected ring currents on the Pt ring electrode, and b) transfer numbers of B,N@C catalysts in 0.1 M KOH.

Table S1. Elemental contents of B, N, and C according to XPS spectra.

Table S2. B and N configurations according to XPS spectra.

Table S3. Comparison of the ORR performance of carbon-based matel-free electrocatalysts in alkaline media (0.1M KOH).

Material Synthesis and Characterization

Chemicals and materials. Zinc nitrate hexahydrate ($Zn(NO_3)_2 \cdot 6H_2O$), cyclohexane, and methanol were purchased from Sinopharm Chemical Reagent Co., Ltd. 2-methylimidazole (2-MIM) was purchased from Aladdin Reagent. Ammonia borane (AB) was purchased from Macklin Co., Ltd. Commercial 20 wt% Pt/C was purchased from Alfa Aesar Chemical Reagent Co., Ltd. Nafion (5 wt %) was purchased from Sigma-Aldrich. All chemicals were of analytical grade and used without further purification. Deionized water was used for all experiments.

Synthesis of ZIF-8. $Zn(NO_3)_2 \cdot 6H_2O$ (3 mmol) was dissolved in 30 mL methanol to form solution A. Solution B contains 24 mmol 2-methylimidazole dissolved in 20 mL methanol. Then, solution B was added to solution A and the mixture was stirred for 24 h at room temperature. The resultant white precipitate was collected by centrifugation, washed with methanol three times, and finally dried overnight.

Synthesis of AB@ZIF-8. In a typical synthesis, 0.1 g of ZIF-8 was added to 20 mL of cyclohexane and ultrasonically treated to form a uniform suspension. Then 0.1 mL of AB aqueous solution (0.1 g/mL) was added into the ZIF-8 dispersion dropwise and stirred for different times (2, 6, 12, 24, and 48 hours). The resultant solids were collected by centrifugation and vacuum dried and were labeled as AB@ZIF-xh, where x is the treatment time.

Synthesis of B,N@C. For carbonization, the obtained AB@ZIF-xh were heated from room temperature to 1000 °C at a heating rate of 5 °C min^{-1} and held at 1000 °C for 2 h under N_2. The obtained samples were denoted as B,N@C-xh.

Materials characterization. Transmission electron microscopy (TEM) images were taken using Hitachi HT7700 operated at an acceleration voltage of 100 kV. The aberration-corrected high-angle annular dark-field scanning transmission electron microscopy (HAADF-STEM) measurements were performed on JEM-ARM200F. Energy-dispersive X-ray spectra (EDS) were collected on JEOL JEMF200. Scanning electron microscope (SEM) images were acquired on JSM-7900F at an accelerating voltage of 2 kV. Nitrogen adsorption-desorption isotherms were collected on Micromeritcs ASAP 2460. The Brunauer–Emmett–Teller (BET) method was used to analyze the specific surface areas. Pore size distributions were

determined by the non-local density functional theory method. X-ray photoelectron (XPS) spectra were obtained on Thermo Scientific K-Alpha using monochrome Al K_α as the excitation light source (hν = 1,486.6 eV). All spectra were calibrated against C 1s peak at the binding energy of 284.8 eV. Wide-angle powder X-ray diffraction (PXRD) was performed on a Rigaku SmartLab diffractometer using Cu K_α radiation (λ = 1.5418 Å) with a voltage of 40 kV and current of 200 mA.

Electrochemical measurements. A PARSTAT 3000A-DX workstation was used to record the electrochemical response at a certain potential. Rotation ring disk electrode (RRDE) measurements were carried out at room temperature using a typical three-electrode system. An Ag/AgCl electrode (saturated KCl) was used as the reference electrode. An RRDE assembly consisting of a glassy carbon rotation disk electrode (s = 0.2475 cm^2) and a Pt ring (s = 0.1866 cm^2) was used with a theoretical collection efficiency of 37%. To prepare a working electrode with a catalyst layer, 5 mg of the as-prepared B,N@C catalyst was mixed with 175 μL of ethanol and 47.5 μL of Nafion 117 solution (5%, Sigma-Aldrich), and sonicated for 20 min to get a homogeneous catalyst ink. 7 μL of the ink was pipetted onto a glassy carbon disk and dried under vacuum.

The kinetic current density (J_K) was calculated using the Koutecky-Levich equation:

$$\frac{1}{J} = \frac{1}{J_L} + \frac{1}{J_K} \quad 1$$

where J is the measured current density, J_K and J_L are the kinetic and limiting current densities.

The transferred electron numbers (n) and H_2O_2 (H_2O_2%) selectivity were calculated based on the disk current (I_d) for O_2 reduction and ring current (I_r) for H_2O_2 oxidation at the Pt ring from RRDE using the following equation.

$$n = \frac{4I_d}{I_d + I_r/N} \qquad 2$$

$$H^2O^2\% = 200\frac{I_r/N}{I_d + I_r/N} \qquad 3$$

Finite-Element Simulation Methods. The finite element analysis (FEA) simulation was conducted using COMSOL Multiphysics. The simulation was implemented in a 2D mode. According to the experimental data, the model of the micropore-dominated material was

constructed as a hexagon about 80 nm on a side with microchannels of 0.9 nm; the model of trimodal-porous materials was plotted with microchannels of 0.9 nm, mesochannels of 7 nm, and cavities of 30 nm inside the hexagon; the model of the macropore-dominated hollow material was constructed with mesochannels of 7 nm in the hexagonal shell about 10 nm thick. These models could effectively simplify the structures of B,N@Cs while maintaining their structural properties.(1-2) A rectangular region with 6000 X 6000 nm^2 was filled with water, which served as the calculated domain. Catalyst was placed in the center of the calculated domain to investigate the velocity fields of nanoarchitecture models. Water was introduced from the left boundary and outflowed from the right. The inflow velocity was set to 0.25 m s^{-1} at the entrance, modelling the flow induced by the rotation of the electrode. The flow field was done on the "Laminar Flow" module. The flow field was solved by the Navier–Stokes equations:

$$\nabla \cdot u = 0 \quad (4)$$

$$\rho(u \cdot \nabla)u = \nabla \cdot [-pI + \mu(\nabla u + (\nabla u)^T)] + F \quad (5)$$

Where u, ρ, p, and F represent the flow velocity, fluid density, pressure, and volume force vector, respectively.

Supporting Figures

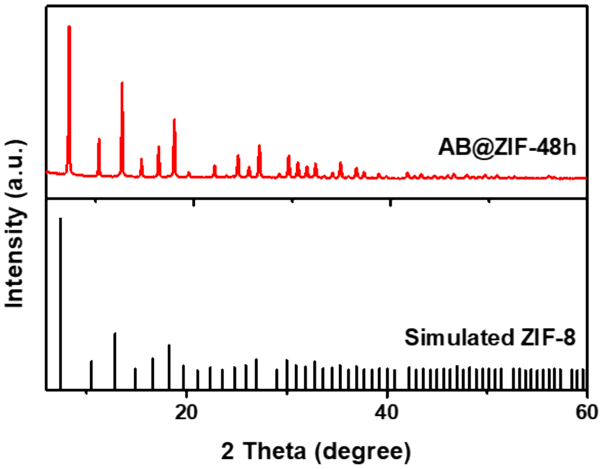

Figure S1. Powder X-ray diffraction (XRD) patterns of the as-synthesized AB@ZIF-8 precursors in comparison with simulated XRD patterns of ZIF-8 based upon single crystal XRD data.

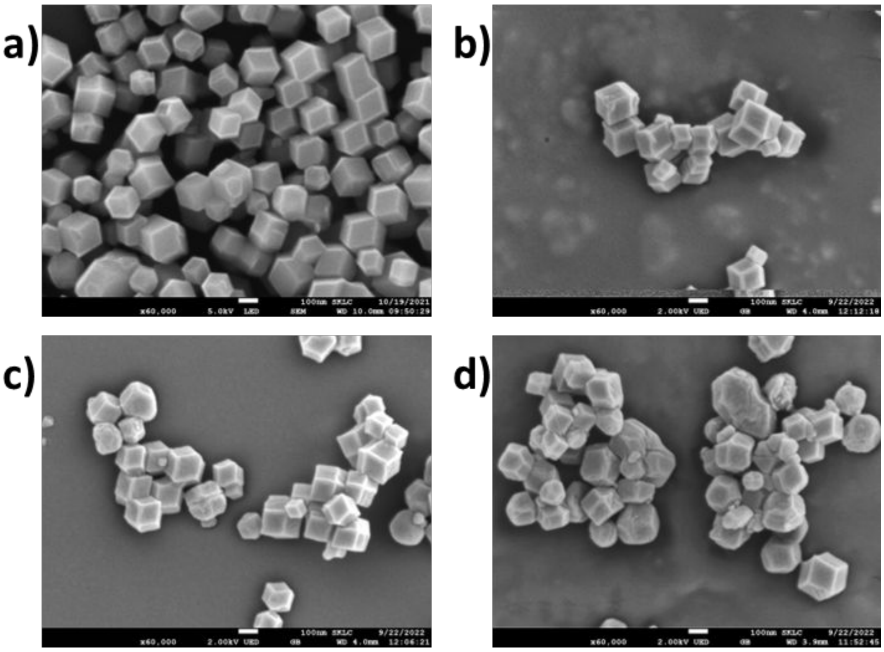

Figure S2. SEM images of a) ZIF-8, b) AB@ZIF-12h, c) AB@ZIF-24h, and d) AB@ZIF-48h.

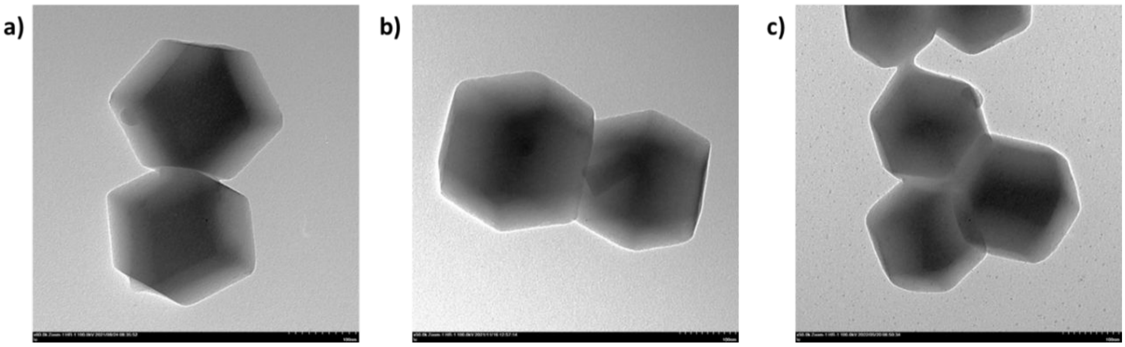

Figure S3. TEM images of a) ZIF-8, b) AB@ZIF-2h and c) AB@ZIF-6h.

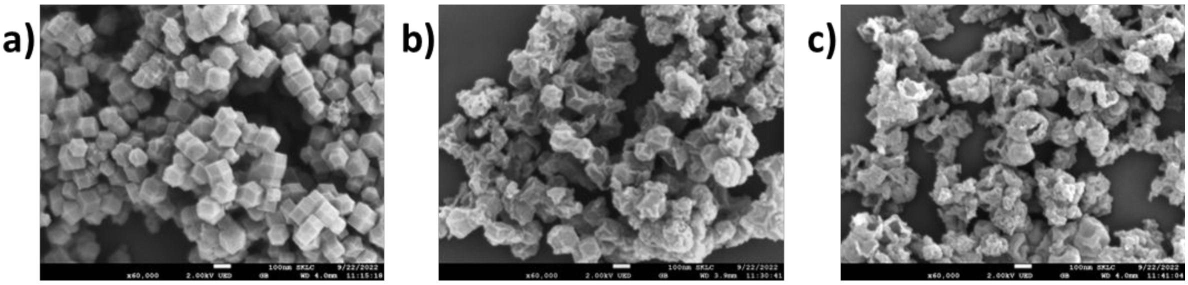

Figure S4. SEM images of a) B,N@C-12h, b) B,N@C-24h, and c) B,N@C-48h.

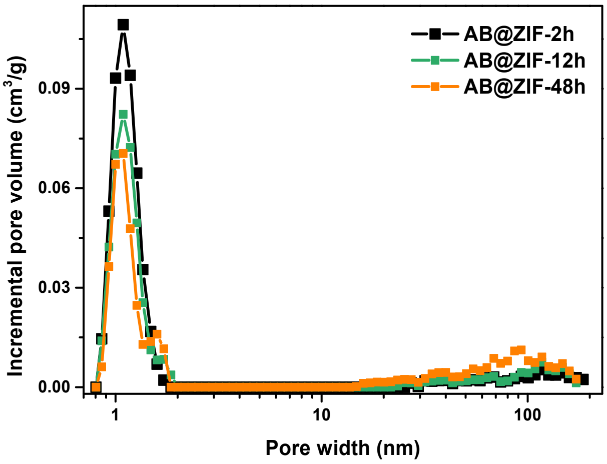

Figure S5. Pore size distribution curves of AB@ZIF-8 precursors.

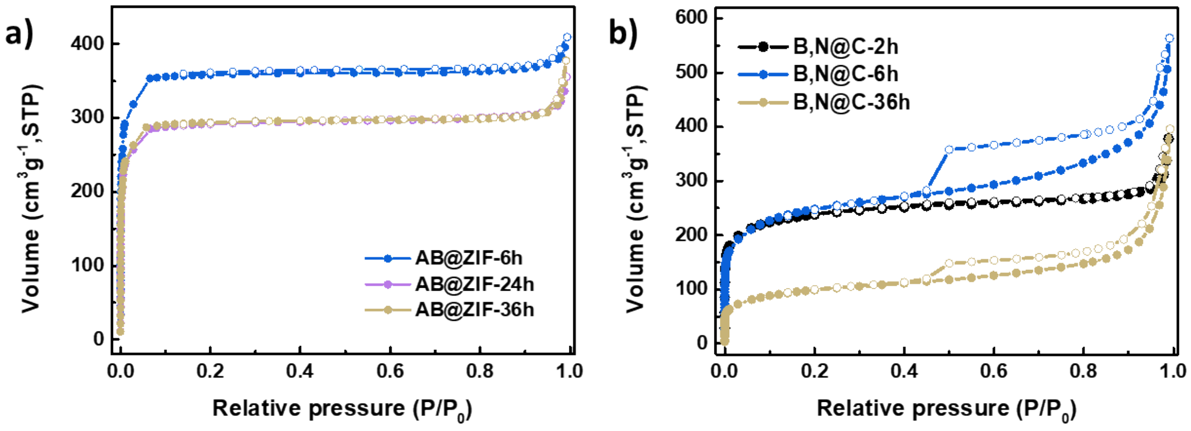

Figure S6. N_2 adsorption-desorption isotherms of a) AB@ZIF-8 and b) B,N@C.

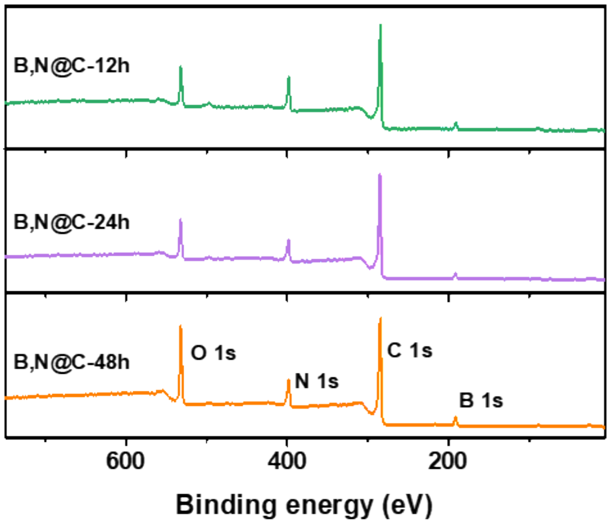

Figure S7. XPS survey spectra of B,N@C.

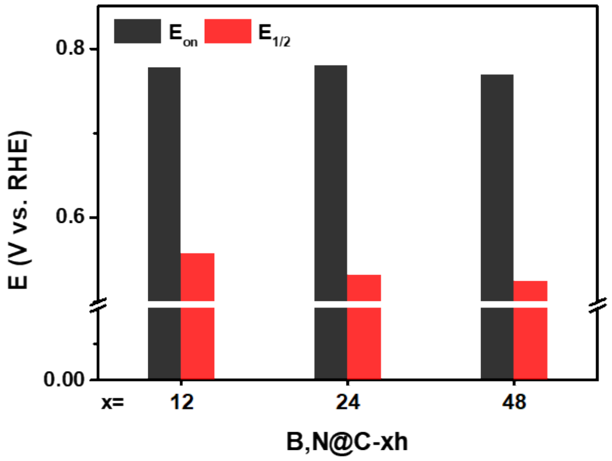

Figure S8. Onset and half-wave potentials of the B,N@C nanoreactors in 0.01 M KOH solution.

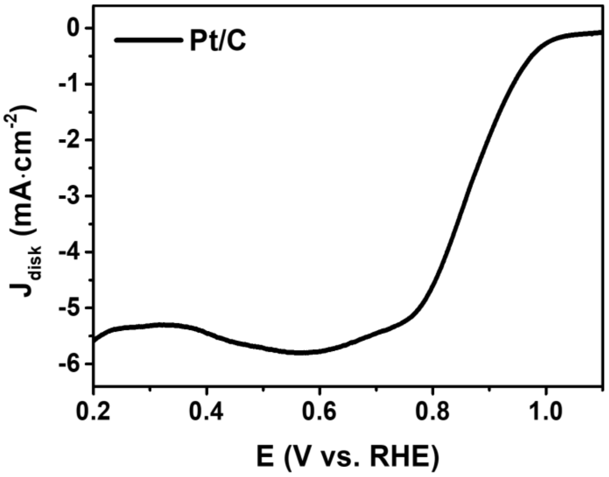

Figure S9. LSV curve of commercial Pt/C in an O_2-saturated electrolyte (0.1 M KOH).

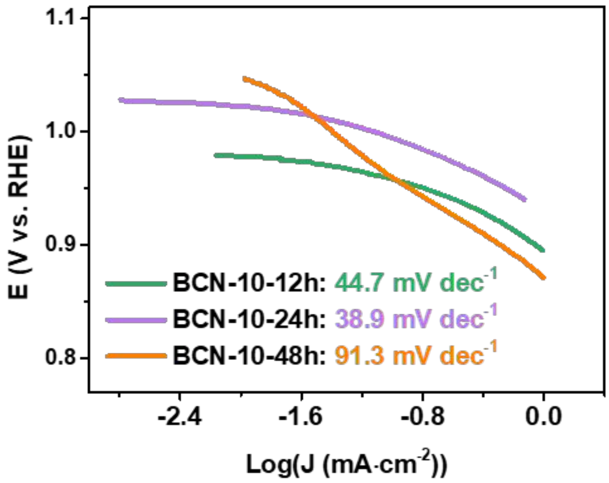

Figure S10. Tafel plots of the B,N@C nanoreactors in O_2-saturated electrolytes (0.1 M KOH).

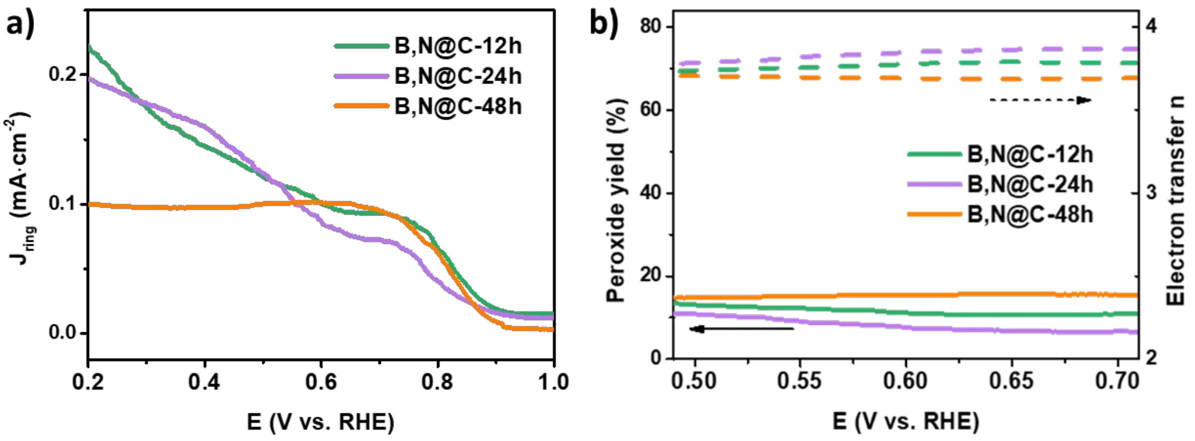

Figure S11. a) The detected ring currents on the Pt ring electrode, and b) transfer numbers of B,N@C catalysts in 0.1 M KOH.

Supporting Tables

Table S1. Elemental contents of B, N, and C according to XPS spectra.

Sample	Elemental content (at.%)			Heteroatomic B and N content (at.%)
	B	C	N	
B,N@C-12h	12.0	72.7	15.3	27.3
B,N@C-24h	9.9	77.6	12.5	22.4
B,N@C-48h	14.2	73.8	12.1	26.3

Table S2. B and N configurations according to XPS spectra.

Sample	Content of B (at.%)			Content of N (at.%)				
	B-C	B-N	B-O	N-B	N6	N5	NQ	N-O
B,N@C-12h	6.0	5.8	0	2.4	6.7	2.3	2.2	1.8
B,N@C-24h	4.7	4.9	0.4	2.0	4.9	2.1	2.1	2.0
B,N@C-48h	5.8	5.2	3.3	2.1	4.6	2.0	2.1	1.4

Table S3. Comparison of the ORR performance for carbon-based matel-free electrocatalysts in alkaline media (0.1M KOH).

Catalysts	$E_{1/2}$ (V vs RHE)	LDCD (mA/cm^2)	Pore structures
B,N@C-24h (this work)	0.979	-5.9	Micro/meso/macropores
B,N@C nanotubes(3)	0.820	-5.5	Mesopores
B,N@ordered macroporous C(4)	0.810	-5.2	Micro/meso/macropores
B,N@ordered mesoporous C(5)	0.748	-5.6	Mesopores
B,N@graphene aerogels(6)	0.785	-5.7	Meso/macropores
Hollow N@C(7)	0.818	-5.3	Meso/macropores
N, S@C nanosphere(8)	0.870	-6.0	Micro/mesopores

Reference

(1) Wang, S.; Zhang, L.; Xia, Z.; Roy, A.; Chang, D. W.; Baek, J.-B.; Dai, L., BCN graphene as efficient metal-free electrocatalyst for the oxygen reduction reaction. *Angew. Chem. Int. Ed.* **2012**, *51* (17), 4209-4212.

(2) Jing, L.; Tang, C.; Tian, Q.; Liu, T.; Ye, S.; Su, P.; Zheng, Y.; Liu, J., Mesoscale diffusion enhancement of carbon-bowl-shaped nanoreactor toward high-performance electrochemical H_2O_2 production. *ACS Appl. Mater. Interfaces* **2021**, *13* (33), 39763-39771.

(3) Wei, P.; Li, X.; He, Z.; Sun, X.; Liang, Q.; Wang, Z.; Fang, C.; Li, Q.; Yang, H.; Han, J.; Huang, Y. Porous N, B co-doped carbon nanotubes as efficient metal-free electrocatalysts for orr and Zn-air batteries. *Chem. Eng. J.* **2021,** 422, 130134.

(4) Li, X.; Fan, L.; Xu, B.; Shang, Y.; Li, M.; Zhang, L.; Liu, S.; Kang, Z.; Liu, Z.; Lu, X.; Sun, D. Single-atom-like B-N_3 sites in ordered macroporous carbon for efficient oxygen reduction reaction. *ACS Appl. Mater. Interfaces* **2021,** 13, (45), 53892-53903.

(5) Zeng, K.; Su, J.; Cao, X.; Zheng, X.; Li, X.; Tian, J.-H.; Jin, C.; Yang, R. B, N co-doped ordered mesoporous carbon with enhanced electrocatalytic activity for the oxygen reduction reaction. *J. Alloys Compd.* **2020,** 824, 153908.

(6) Chen, W.; Xu, L.; Tian, Y.; Li, H.; Wang, K. Boron and nitrogen co-doped graphene aerogels: Facile preparation, tunable doping contents and bifunctional oxygen electrocatalysis. *Carbon* **2018,** 137, 458-466.

(7) Chai, L.; Zhang, L.; Wang, X.; Xu, L.; Han, C.; Li, T.-T.; Hu, Y.; Qian, J.; Huang, S. Bottom-up synthesis of MOF-derived hollow n-doped carbon materials for enhanced ORR performance. *Carbon* **2019,** 146, 248-256.

(8) Zhang, X.; Yao, S.; Chen, P.; Wang, Y.; Lyu, D.; Yu, F.; Qing, M.; Tian, Z. Q.; Shen, P. K. Revealing the dependence of active site configuration of N doped and N, S-co-doped carbon nanospheres on six-membered heterocyclic precursors for oxygen reduction reaction. *J. Catal.* **2020,** 389, 677-689.
